# Feeding Intolerance in Critically Ill Patients with Enteral Nutrition: A Meta-Analysis and Systematic Review

**DOI:** 10.2478/jccm-2024-0007

**Published:** 2024-01-30

**Authors:** Jing Xu, Wenyu Shi, Liying Xie, Jing Xu, Lanzheng Bian

**Affiliations:** Department of Cardiothoracic surgery, Children’s Hospital of Nanjing Medical University, Nanjing, China; Department of Nursing, Children’s Hospital of Nanjing Medical University, Nanjing, China

**Keywords:** feeding intolerance, critically ill, enteral nutrition, care, management, nursing

## Abstract

**Background:**

Feeding intolerance is a common yet serious complication in critically ill patients undergoing enteral nutrition. We aimed to conduct a meta-analysis to evaluate the risk factors of feeding intolerance in critically ill patients undergoing enteral nutrition, to provide insights to the clinical enteral nutrition treatment and care.

**Methods:**

Two researchers systematically searched PubMed, Medline, Web of Science, Cochrane Library, Chinanews. com, Wanfang and Weipu databases about the studies on the risk factors of feeding intolerance in severe patients with enteral nutrition up to August 15, 2023. Literature screening, data extraction and quality evaluation were carried out independently by two researchers, and Meta analysis was carried out with RevMan 5.3 software and Stata 15.0 software.

**Results:**

18 studies involving 5564 enteral nutrition patients were included. The results of meta-analyses showed that age < 2 years old, age > 60 years old, APACHE II score ≥ 20, Hypokalemia, starting time of enteral nutrition > 72 hours, no dietary fiber, intra-abdominal pressure > 15mmHg, central venous pressure > 10cmH_2_O and mechanical ventilation were the risk factors of feeding intolerance in critically ill patients undergoing EN (all P<0.05). No publication biases were found amongst the included studies.

**Conclusion:**

The incidence of feeding intolerance in critically ill patients undergoing enteral nutrition is high, and there are many influencing factors. Clinical medical workers should take effective preventive measures according to the risk and protective factors of patients to reduce the incidence of feeding intolerance and improve the prognosis of patients.

## Introduction

Critically ill patients are in a state of high metabolism due to trauma, surgery, infection and stress, which will lead to uneven release of cytokines and stress hormones, thus changing the metabolism of energy and protein, leading to malnutrition [1]. Previous studies [2, 3] has shown that the incidence of malnutrition in intensive care unit (ICU) patients is 38%~78%. Malnutrition has been shown to be independently associated with increased mortality, length of stay and hospital costs. Therefore, it is very important to give proper nutritional support to critically ill patients [4]. The American Society for Parenteral and Enteral Nutrition (ASPEN) [5] has proposed enteral nutrition (EN) as the first choice of nutritional support for critically ill patients, and has recommended that critically ill patients should undergo EN within 24 to 48 hours after admission to ICU without EN contraindications. Early EN can meet the demand for nutrients, maintain the function of various organs, reduce infection rate and complications, and promote the recovery of neurological function and reduce mortality.

Although early enteral nutrition can effectively improve the nutritional status of critically ill patients. However, during EN, patients often suffer from feeding intolerance such as diarrhea, abdominal distension, nausea and vomiting, gastric retention and so on [6]. The incidence of EN feeding intolerance is 30.5% ~ 65.7%, which leads to the suspension of EN and the inability of patients to absorb sufficient energy and nutrients, which seriously affects the nutritional status of patients [7, 8]. Therefore, how to avoid the occurrence of EN feeding intolerance should be paid more attention by clinical medical staff. At present, there are different reports on the influencing factors of EN feeding intolerance in critically ill patients. The purpose of this study was to analyze the risk factors of EN feeding intolerance in critically ill patients by meta-analysis, in order to provide evidence support and reference for health care providers to prevent and manage EN feeding intolerance.

## Methods

This study was conducted and reported in accordance to the preferred reporting items for systematic reviews and meta-analyses (PRISMA) statement [9]. Ethical approval and informed consents were not necessary since the study was a meta-analysis.

### Literature retrieval strategy

The two researchers systematically searched PubMed, Medline, Web of Science, Cochrane Library, Chinanews.com, Wanfang and Weipu databases about the studies on the risk factors of feeding intolerance in severe patients with enteral nutrition. The time range of retrieval was from the establishment of the database to August 15, 2023. In this study, the literature retrieval strategies were as following: (“enteral nutrition” OR “enteral feeding” OR “feeding intolerance”) And (“factors” OR “risk factors” OR “influence factor”). We traced the references to the included literature in order to reduce the possibility of missing reports.

### Inclusion and exclusion criteria

The inclusion criteria of this meta-analysis were as follows: Study population: severe patients who received enteral nutrition support, the population and gender were not limited. Exposure factors: patients’ enteral nutrition feeding intolerance may be related to factors such as age, mechanical ventilation, etc. Outcome indicators: risk factors of EN feeding intolerance. Study types: cohort study or case-control study. The exclusion criteria of this meta-analysis were as follows: The literature whose data could not be extracted for reasons such as incomplete and incorrect data; The studies that were unable to obtain the full text; repeatedly published literature; case reports, reviews and other conference literatures.

### Data extraction and collection

The retrieved literature was firstly deduplicated by End-Note software. And then the remaining titles and abstracts are read independently by two evaluators, and the full text is re-screened according to the inclusion and exclusion criteria of the literature. The third party decided whether or not to include the controversial literature in the process of literature screening. Following data were extracted and collected: the first author, the year of publication, the type of study design, the total sample size, the sample size of the case group, exposure factors.

### Literature quality evaluation

Two researchers used the Newcastle-Ottawa Scale (NOS) to evaluate the quality of the included literature. The NOS scale included three aspects: the selection of the study population, the comparability between groups and the evaluation of exposure, with a total of 8 items. And the full score of NOS was 9. Literature screening, data extraction and literature quality evaluation were carried out independently by two researchers and cross-checked. In case of differences, we resolved them through consultation with the third researcher.

### Statistical method

This meta-analysis used RevMan5.3 and Stata 15.0 software to merge and analyze the extracted data. The odds ratio (OR) was used as the effect statistic, and the inter-regional estimation was expressed by 95% confidence interval (95% CI). Firstly, the heterogeneity test was carried out, if the heterogeneity was small (P ≥ 0.1, I^2^ ≤ 50%), the fixed effect model was used for combined analysis, and if the heterogeneity among the studies was large (P < 0.1, I^2^ > 50%), random effect model was used for combined analysis. Sensitivity analysis was carried out by changing the data analysis model to test the stability of Meta results. Publication bias was analyzed by funnel chart and Egger test. In this study, the difference between groups was statistically significant when P < 0.05.

## Results

### Study inclusion

**Fig. 1.**
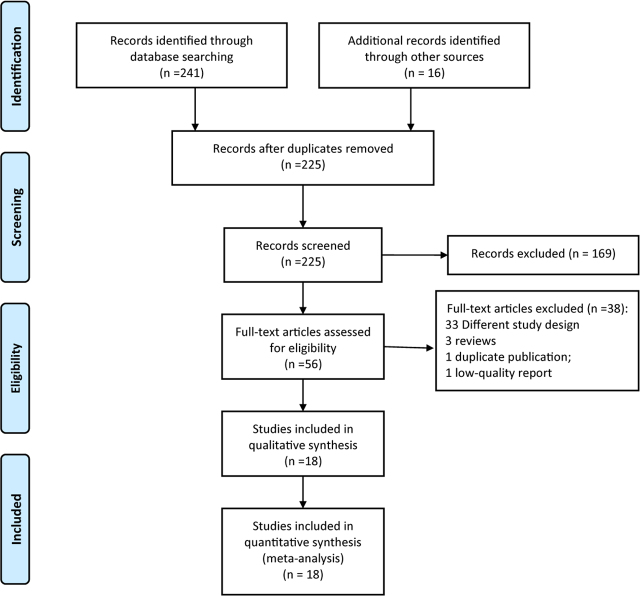
The flow chart of study selection

A total of 257 articles were initially searched. 702 literatures were obtained after duplicate removal. 225 reports were included after the initial selection. Based on the inclusion and exclusion criteria, we finally included 18 studies [10,11,12,13,14,15,16,17,18,19,20,21,22,23,24,25,26,27], involving 5564 EN patients. The literature screening process is shown in Figure 1.

### Characteristics and quality of included studies

Among the 18 articles included, 14 were case-control studies and 4 were cohort studies, with a total of 5564 patients. The incidence of EN feeding intolerance is 29.17% ~ 60.47%. The basic characteristics of the literature are shown in Table 1. The NOS score scores of the included literatures were all ≥ 7, and the overall quality of the included literatures was good. The evaluation results of literature quality are shown in Table 2.

**Table 1:** The characteristics of included studies

**Study**	**Design**	**Sample size**	**Case/control**	**Incidence of feeding intolerance**	**Influencing factors**
Bejarano 2012	Cohort study	82	21/51	29.17%	2, 6, 12
Chen 2017	Case control study	92	54/38	58.69%	2, 4, 6, 7, 8, 9, 11, 12, 13
Cai 2018	Case control study	373	155/218	41.55%	1, 3, 7, 8, 10, 12, 13, 14
Sun 2018	Case control study	295	121/174	41.02%	3, 6, 9, 10, 12, 15, 16
Liu 2018	Case control study	86	52/34	60.47%	3, 4, 5, 7, 10, 13
Jin 2018	Case control study	243	84/159	34.57%	1, 3, 6, 7, 8, 9, 10, 11, 12, 13, 15
Wang 2019	Case control study	118	49/69	41.53%	3, 4, 5, 9, 11
Zhou 2019	Case control study	84	33/51	39.29%	3, 4, 6, 9, 11, 13, 16
Liu 2020	Case control study	200	115/85	57.50%	1, 7, 9, 10
Deng 2020	Case control study	120	68/52	56.67%	3, 12, 13
Zhai 2020	Case control study	91	49/42	53.85%	3, 4, 5, 6, 9, 10, 12, 13, 14
Li 2019	Case control study	568	184/384	32.39%	3, 6, 16
Mentec 2001	Cohort study	153	59/94	38.56%	3, 4, 5, 13, 15
Gungabissoon 2014	Cohort study	1888	576/1312	30.51%	4, 6, 9
Li 2021	Case control study	70	29/41	41.43%	1, 4, 5, 6, 10
Geng 2022	Case control study	884	352/532	39.82%	1, 6, 7, 9, 12
Zhang 2022	Cohort study	83	36/47	43.37%	2, 4, 5, 8, 10, 13
Zheng 2021	Case control study	134	56/78	41.79%	2, 5, 7, 9

Notes: 1: age > 60 years old; 2: age < 2 years old; 3: male; 4: acute physiological function and chronic health score II (APACHE II) score ≥ 20; 5: hyperglycemia; 6: hypokalemia; 7: hypoproteinemia; 8: starting time of enteral nutrition > 72 hours; 9: no dietary fiber; 10: intra-abdominal pressure > 15mmHg (1mmHg=0.133kPa); 11: central venous pressure > 10cmH_2_O(cmH_2_O0.098kPa); 12: mechanical ventilation; 13: use of sedatives; 14: use of vasoactive drugs; 15: use of acidogenic agents; 16: blood purification.

**Table 2:** The NOS score of included studies

**Study**	**Patient selection**	**Comparability**	**Exposure assessment**	**NOS total score**
Bejarano 2012	3	2	2	7
Chen 2017	3	2	2	7
Cai 2018	3	2	2	7
Sun 2018	3	2	3	8
Liu 2018	3	2	2	7
Jin 2018	3	2	2	7
Wang 2019	3	2	3	8
Zhou 2019	3	2	2	7
Liu 2020	3	2	2	7
Deng 2020	3	1	3	7
Zhai 2020	3	2	2	7
Li 2019	3	2	3	8
Mentec 2001	3	2	2	7
Gungabissoon 2014	3	2	2	7
Li 2021	3	2	3	8
Geng 2022	3	2	3	8
Zhang 2022	3	2	3	8
Zheng 2021	3	2	2	7

## Meta-analysis

As indicated in Table 3, the results of meta-analyses showed that age < 2 years old, age > 60 years old, APACHE II score ≥ 20, Hypokalemia, starting time of enteral nutrition > 72 hours, no dietary fiber, intra-abdominal pressure > 15mmHg, central venous pressure > 10cmH_2_O and mechanical ventilation were the risk factors of feeding intolerance in critically ill patients undergoing EN (all P<0.05). Hyperglycemia, hypoproteinemia, use of sedatives, use of vasoactive drugs, use of acidogenic agents and blood purification were not associated with the feeding intolerance in critically ill patients undergoing EN (all P>0.05).

**Table 3:** The meta-analysis on the risk factors of feeding intolerance in critically ill patients undergoing enteral nutrition

**Factors**	**Number of synthesized studies**	**Heterogeneity**		**Model**	**Synthesized effects**		
		I^2^(%)	P		OR	95%CI	P
Age < 2 years old	2	16	0.44	Fixed	1.84	1.32~2.05	0.015
Age > 60 years old	4	0	0.75	Fixed	2.04	1.45~2.61	0.007
APACHE II score ≥ 20	6	11	0.18	Fixed	3.18	2.95~3.89	0.033
Hyperglycemia	4	59	0.03	Random	1.70	0.88~2.46	0.069
Hypokalemia	5	47	0.11	Fixed	1.59	1.24~2.03	<0.001
Hypoproteinemia	5	89	<0.01	Random	0.70	0.26~1.90	0.486
Starting time of enteral nutrition > 72 hours	3	0	0.87	Fixed	2.24	1.49~3.36	<0.001
No dietary fiber	8	68	0.01	Random	3.72	2.23~6.19	<0.001
Intra-abdominal pressure > 15mmhg	7	0	0.51	Fixed	2.95	2.10~3.85	<0.001
Central venous pressure > 10cmh_2_o	4	45	0.15	Fixed	1.96	1.47~2.62	<0.001
Mechanical ventilation	9	44	0.23	Fixed	1.37	1.19~1.89	0.012
Use of sedatives	7	74	<0.01	Random	1.19	0.93~1.53	0.161
Use of vasoactive drugs	3	89	<0.01	Random	0.95	0.56~1.61	0.854
Use of acidogenic agents	3	0	0.41	Fixed	0.84	0.52~1.38	0.506
Blood purification	4	97	<0.01	Random	1.41	0.86~2.30	0.171

APACHE II: acute physiological function and chronic health score II

### Publication bias and sensitivity analysis

Publication bias assessment and sensitivity analysis found that the funnel diagrams of each index were basically symmetrical. The publication bias of the included literature was analyzed by Egger’s test. There was no significant publication bias in the all 14 exposure factors (all P > 0.05, Table 4). Through the fixed effect model and random effect mode conversion to analyze the sensitivity of the combined effect, it is found that the difference between the two combined effects was small, indicating that the results of meta-analysis were stable.

**Table 4:** Egger’s test results for detecting publication bias of included studies

**Factors**	**Egger’s test**	
	**t**	**P**
Age < 2 years old	1.88	0.209
Age > 60 years old	2.47	0.113
APACHE II score ≥ 20	1.14	0.265
Hyperglycemia	−1.32	0.179
Hypokalemia	5.30	0.013
Hypoproteinemia	−0.56	0.612
Starting time of enteral nutrition > 72 hours	−1.88	0.312
No dietary fiber	1.89	0.131
Intra-abdominal pressure > 15mmhg	1.18	0.965
Central venous pressure > 10cmh_2_o	3.54	0.971
Mechanical ventilation	2.08	0.331
Use of sedatives	−0.22	0.832
Use of vasoactive drugs	−0.14	0.910
Use of acidogenic agents	10.58	0.060
Blood purification	−0.16	0.572

APACHE II: acute physiological function and chronic health score II.

## Discussions

Enteral nutrition is preferred in critically ill patients, which is recommended and applied by many clinical guidelines at present [28]. Low-dose enteral nutrition can start safely within 48 hours after admission, even during low-or medium-dose vasopressor treatment [29]. Energy delivery should not be calculated to match energy consumption until 4–7 days, and the use of high-energy formulations can be limited to patients who cannot tolerate full-capacity enteral nutrition or require fluid restriction to avoid over feeding syndrome [30]. A low dose of protein (maximum 0.8g/kg/d) can be provided in the early stages of critical illness, while the protein goal of > 1.2g/kg/d can be considered in the rehabilitation phase [31]. The occurrence of refeeding syndrome should be assessed by daily determination of blood phosphorus, and a 30% reduction in phosphorus should be controlled by reducing enteral feeding rates and high doses of thiamine [32]. Vomiting, increased gastric residue, abdominal pain and abdominal distension may indicate gastrointestinal intolerance [33]. It has been reported that gastrointestinal complications are the most common problems of enteral nutrition, but the appropriate selection of formula, route, feeding mode, administration time and dose can reduce the risk of these complications [34]. Therefore, it is of great significance to understanding the influencing factors of feeding intolerance for the treatment and nursing of critically ill patients.

Age < 2 years old or > 60 years old is a risk factor for enteral nutrition intolerance in critically ill patients. The gastrointestinal function of young children is not fully developed, and feeding intolerance is easy to occur [35, 36]. Previous meta-analysis [37] has also shown that age > 60 years old was a risk factor for enteral nutrition intolerance in critically ill patients. In the elderly, esophageal sphincter relaxation is prone to symptoms such as food reflux and gastric retention [38]. Due to the decrease of gastrointestinal function in elderly patients and the severity of the disease, most patients stay in bed for a long time and reduce their independent activity, resulting in poor gastrointestinal peristalsis, which slows down gastric emptying and is not conducive to the intake of EN [28, 39]. Therefore, for patients less than 2 years old or more than 60 years old, nurses should fully evaluate their gastrointestinal function and formulate a reasonable and effective nutrition intervention plan.

High APACHE II score is a risk factor for EN feeding intolerance. APACHE II score is an important index to evaluate the severity of the disease. The results of previous studies [40, 41] showed that patients with high APACHE II score were prone to EN feeding intolerance, which was consistent with the results of this study. This is due to the high APACHE II score, which indicates that the patient’s condition is serious and the stress response of the body is enhanced, which leads to the serious damage of gastrointestinal function and the deterioration of gastrointestinal tolerance. Therefore, during EN, this kind of patients cannot tolerate the infusion of nutrient solution and have corresponding gastrointestinal complications. For the patients with high APACHE II score, especially those with a score of more than 20, nurses should strengthen the monitoring in the process of EN, timely evaluate whether the patients show signs of feeding intolerance, and avoid or reduce the occurrence of EN feeding intolerance as far as possible.

High levels of intra-abdominal pressure can cause EN feeding intolerance in critically ill patients. This is because the increase in intra-abdominal pressure will lead to a decrease in gastrointestinal blood flow, resulting in gastrointestinal integrity damage, affecting gastrointestinal function. It’s been reported that adjusting the speed of EN infusion according to the level of intra-abdominal pressure can prevent the occurrence of EN intolerance [42, 43]. However, because the measurement of intra-abdominal pressure is affected by artificial subjective judgment, mechanical ventilation and other factors, there are often some errors, and nurses do not pay enough attention to the monitoring of patients’ intra-abdominal pressure and lack of relevant theoretical knowledge, which indirectly leads to EN feeding intolerance [44, 45]. Therefore, the training of nurses on intra-abdominal pressure monitoring should be strengthened to ensure the effectiveness and accuracy of patients’ intra-abdominal pressure monitoring.

The starting time of enteral nutrition > 72 hours, no dietary fiber and mechanical ventilation were the risk factors of enteral feeding intolerance in critically ill patients. The starting time of enteral nutrition > 72 hours is significantly related to enteral nutrition feeding intolerance. Early fasting in critically ill patients can avoid stimulating pancreatic juice secretion and promote intestinal rest, but with the extension of fasting time, gastrointestinal self-repair ability and bactericidal ability decreased, resulting in intestinal function disorder, resulting in enteral nutrition intolerance [46, 47]. Early enteral nutrition helps to protect intestinal mucosal barrier function and reduce bacterial translocation, thus reducing the incidence of complications such as infection. The relevant guidelines indicate that severe patients should start enteral nutrition 24–72 hours after admission. The fermentation of dietary fiber in the colon can produce short-chain fatty acids, which can improve intestinal immune function and regulate gastrointestinal motility [48]. At the same time, it can prolong the transit time of food in the gastrointestinal tract, reduce the occurrence of diarrhea, and then improve the tolerance of enteral nutrition [49]. Some studies [50, 51] have shown that the addition of dietary fiber to enteral nutrition in critically ill patients can reduce inflammatory response, improve immune response and correct intestinal mucosal barrier dysfunction. At present, the guidelines at home and abroad have not clearly pointed out that dietary fiber should be added to enteral nutrition preparations for severe patients. The effect of dietary fiber on enteral nutrition tolerance in patients with severe acute pancreatitis has yet to be confirmed by multicenter, large sample, high-quality clinical studies.

Previous study [52] has reported that the incidence of feeding intolerance in mechanically ventilated patients is as high as 50.8% to 88.9%. Mechanical ventilation will cause a decrease in the supply of gastrointestinal blood fluid, resulting in anoxia and ischemia of gastrointestinal mucosa, resulting in damage to the gastrointestinal function. In addition, mechanical ventilation will increase the intra-abdominal pressure and cause the disorder of gastrointestinal function of the patient. Therefore, enteral nutrition intolerance is easy to occur in patients undergoing mechanical ventilation [53]. Therefore, for patients with mechanical ventilation, it is necessary to closely observe the gastrointestinal tolerance, evaluate respiratory function in time, and stop mechanical ventilation treatment as soon as possible for patients with spontaneous respiratory function recovery, so as to reduce the occurrence of enteral nutrition feeding intolerance.

Some limitations of this study are worthy of careful consideration. First of all, this study only included the published Chinese and English literature, but not the grey literature, so there may be publication bias. Secondly, there are few reports on the factors such as hyperglycemia, the use of vasoactive drugs, the use of acid-making agents and other factors included in the analysis, which may have an impact on the reliability of synthesized outcomes. Thirdly, due to the differences in the criteria for judging EN feeding intolerance in different studies, there is a certain heterogeneity among the studies. Therefore, the results of this study still need to be further demonstrated by more large samples and high-quality prospective studies.

## Conclusions

In conclusion, this meta-analysis has found that age < 2 years old, age > 60 years old, APACHE II score ≥ 20, Hypokalemia, starting time of enteral nutrition > 72 hours, no dietary fiber, intra-abdominal pressure > 15mmHg, central venous pressure > 10cmH_2_O and mechanical ventilation are the risk factors of feeding intolerance in critically ill patients undergoing EN. There are many influencing factors on the risk factors of enteral nutrition intolerance in critically ill patients. Clinical doctors and nurses should timely identify the risk factors of enteral nutrition intolerance in critically ill patients and take positive and effective prevention and treatment measures. and then reduce the incidence of enteral nutrition intolerance, achieve the target feeding volume as soon as possible, and promote the recovery of patients.

## List of abbreviations

ICU: intensive care unit
ASPEN: American Society for Parenteral and Enteral Nutrition
EN: enteral nutrition
PRISMA: preferred reporting items for systematic reviews and meta-analyses
NOS: Newcastle-Ottawa Scale
OR: ratio ratio
95% CI: 95% confidence interval
APACHE II: acute physiological function and chronic health score II.
